# Unilateral Gynecomastia and Primary Hypogonadism Following Brucellosis Infection: A Rare Case Report

**DOI:** 10.1002/ccr3.70880

**Published:** 2025-09-08

**Authors:** Yaghoub Safdari

**Affiliations:** ^1^ Golestan Research Center of Gastroenterology and Hepatology Golestan University of Medical Sciences Gorgan Iran

**Keywords:** brucellosis, FSH, gynecomastia, LH, testosterone

## Abstract

Brucellosis is a bacterial infection with a wide spectrum of clinical manifestations, yet endocrine complications such as primary hypogonadism and gynecomastia are exceedingly rare. We present the case of a 60‐year‐old male who was treated for brucellosis and, several weeks after discharge, developed unilateral enlargement of the left breast. Laboratory evaluation revealed significantly elevated gonadotropins (FSH and LH) with markedly decreased serum testosterone levels, indicating primary testicular failure. Sonographic imaging confirmed gynecomastia with a flame‐shaped, irregular mass. This case highlights a rare hormonal sequela of brucellosis and emphasizes the importance of endocrine evaluation in patients who present with atypical manifestations following systemic infections.

## Graphical abstract

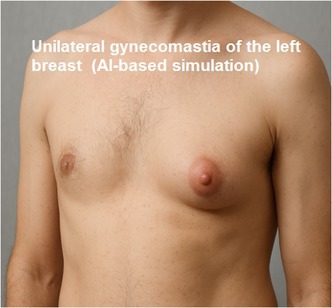

**Summary**

- Gynecomastia following hypogonadism can rarely occur in brucellosis infection.

## 1 Introduction

Brucellosis is a globally prevalent zoonotic disease, primarily caused by *Brucella melitensis* , and transmitted through direct contact with infected animals or ingestion of unpasteurized dairy products [1, 2]. While the infection commonly targets the reticuloendothelial system, it can present with a wide range of manifestations including musculoskeletal, neurological, and genitourinary involvement [2]. Endocrine manifestations of brucellosis are rare, and there is limited literature describing its impact on the hypothalamic–pituitary–gonadal axis [3, 4]. Gynecomastia, the benign proliferation of male breast tissue, typically occurs due to hormonal imbalance—most often a decrease in androgen levels relative to estrogen [5, 6]. This report describes an unusual case of unilateral gynecomastia and primary hypogonadism in a male patient after recovery from brucellosis, highlighting a potential but underrecognized complication [3, 7].

## 2 Case History/Examination/ Differential Diagnosis, Investigations and Treatment

A 60‐year‐old male was diagnosed with brucellosis based on a positive Wright test (see Data S1 and S2) and clinical features including prolonged fever and malaise. He was hospitalized for 3 weeks and treated with appropriate antibiotic therapy. Rifampicin and Doxycycline were the antibiotics that the patient was administered both prior to and following hospital discharge. Following discharge, the patient continued outpatient follow‐up.

**FIGURE 1:**
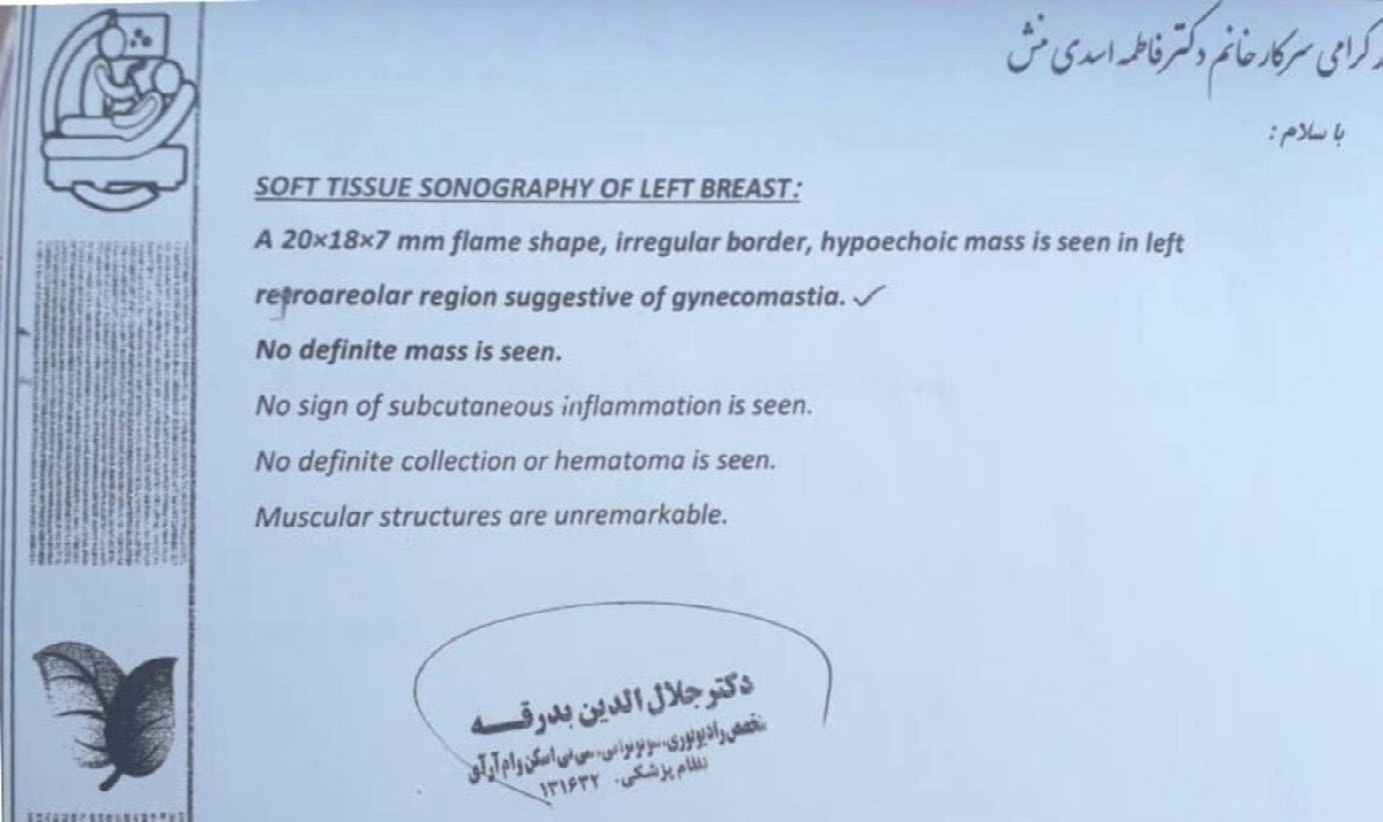
Sonography report of the patient's left breast. The characteristics of the breast mass are shown in the figure.

Approximately 2 months after being discharged from the hospital, breast enlargement and gynecomastia began to manifest. Physical examination revealed soft tissue growth on the left breast without tenderness. Breast sonography revealed a flame‐shaped mass with irregular margins measuring 20 × 1.8 × 7 mm in the left breast, consistent with gynecomastia. No masses or abnormalities were detected in the right breast (Figure 1). The patient did not experience testicular inflammation (orchitis) during the course of the illness. Hormonal profiling is shown in Table 1.

**TABLE 1:** Hormonal test of blood serum.

Hormone	Measured value	Reference range	Status	Unit
FSH	34.1	1.5–12.4	Elevated	mIU/mL
LH	26.9	1.7–8.27	Elevated	mIU/mL
Total testosterone	2.15	2.41–8.27	Low	ng/mL
Free testosterone	3.1	5.9–27	Significantly low	pg/mL

*Note:* All hormone levels were assessed via serum analysis.

Elevated gonadotropins (FSH and LH) alongside reduced testosterone levels are consistent with primary testicular failure.

The hormonal profile was consistent with primary hypogonadism, characterized by high gonadotropins and low testosterone. No history of exposure to endocrine disruptors, liver disease, renal dysfunction, or medications known to cause gynecomastia was reported.

This patient's PSA profile (Table 2) supports a non‐neoplastic origin of hormonal imbalance, reinforcing the hypothesis that gynecomastia was not caused by excess estrogen production from a prostate‐related tumor. Instead, the findings align with primary hypogonadism secondary to testicular dysfunction. PSA levels were within the reference range, excluding prostate‐related estrogen excess.

**TABLE 2:** PSA assay in blood serum.

Test	Value	Reference range	Interpretation
Total PSA	1.13 ng/mL	0–4.0 ng/mL (age‐adjusted)	Within normal limits
Free PSA	0.291 ng/mL	Varies	Absolute value—not interpreted alone
Free PSA ratio	25.8%	> 25% favorable	Suggestive of benign status (e.g., BPH or normal prostate)

*Note:* The Free PSA ratio (25.8%) was calculated using the following formula: (Free PSA/Total PSA) × 100%.

Abbreviation: PSA, prostate‐specific antigen.

Ratios above 25% are commonly associated with benign prostatic conditions or normal function, whereas values below 10% may suggest increased risk of prostate malignancy [8].

## 3 Conclusions and Results

This case report presents a rare endocrine complication of brucellosis in a 60‐year‐old male, manifesting as unilateral gynecomastia and primary hypogonadism. The hormonal profile—markedly elevated LH and FSH with low total and free testosterone—suggests Leydig cell dysfunction, likely secondary to testicular involvement by *Brucella* infection. Sonographic evidence of breast tissue proliferation further supports the clinical diagnosis. Although genitourinary complications such as orchitis are known in brucellosis, endocrine sequelae like hypogonadism and gynecomastia are seldom reported. This case underscores the importance of hormonal evaluation in patients recovering from systemic infections who present with atypical symptoms. Early recognition and appropriate endocrine workup may prevent misdiagnosis and guide timely management.

## 4 Discussion

Brucellosis is a multi‐systemic infection, but endocrine complications remain underreported [1, 3]. This case illustrates an uncommon manifestation—unilateral gynecomastia associated with primary hypogonadism—in a patient recently treated for brucellosis [3, 7]. Laboratory data revealed elevated gonadotropins and low serum testosterone, consistent with testicular failure [5, 6]. The elevated LH and FSH levels suggest that the pituitary is functioning properly and attempting to stimulate testosterone production, but the testes are unable to respond, indicating Leydig cell dysfunction [5]. Importantly, the prostate was evaluated as a potential source of excess estrogen. Total PSA was within normal limits, and the Free PSA ratio was favorable—excluding a prostate‐derived estrogenic cause of gynecomastia. The likely mechanism involves testosterone deficiency after testicular damage, possibly due to immune‐mediated or inflammatory sequelae of brucellosis [1, 4]. This hormonal imbalance favors estrogenic activity, leading to proliferation of breast tissue [5, 6]. While testicular involvement in brucellosis has been documented—such as orchitis and epididymitis—hormonal consequences like gynecomastia are seldom recognized [1, 9]. This case supports the hypothesis that *Brucella* infection may impair steroidogenesis even in the absence of overt gonadal inflammation [4, 9].

More than 2 years have passed since the patient was discharged from the hospital, and the Wright test for brucellosis has remained negative (see Data S3 for laboratory evidences), confirming resolution of the active infection. However, the patient continues to require periodic testosterone injections to maintain hormone levels within the physiological range. This ongoing need indicates that the testicular dysfunction resulting from the infection has become permanent. Given the prolonged course, absence of antibiotic therapy, and persistent endocrine impairment, the clinical presentation is most consistent with chronic brucellosis. The sustained hypogonadism, despite negative serological findings, suggests irreversible damage to the testicular tissue—particularly the Leydig cells responsible for testosterone production. Therefore, the observed hormonal deficiency is more likely due to direct structural or functional impairment caused by the chronic infection, rather than incomplete pharmacological treatment or residual disease activity.

Complementing this endocrine profile, follow‐up assessments revealed no clinical signs of orchitis during the illness, yet breast enlargement and gynecomastia emerged approximately 2 months after discharge. Although gynecomastia may result from hormonal disturbances potentially associated with suboptimal treatment, the present report does not aim to elucidate the molecular mechanisms involved. Instead, it underscores the possibility of gynecomastia occurring in the context of brucellosis, irrespective of the exact pathophysiological pathway. This observation may be of clinical relevance, particularly in cases presenting with atypical manifestations. This condition gradually resolved following the initiation of testosterone replacement therapy, further supporting the hypothesis that the gynecomastia was hormonally mediated.

Clinicians should be aware of this rare but significant endocrine complication, and consider hormonal assessment in post‐brucellosis patients presenting with atypical signs. Further research may clarify the pathophysiology and frequency of such outcomes.

## Author Contributions

**Yaghoub Safdari:** conceptualization, investigation, supervision, validation, writing – original draft, writing – review and editing.

## Ethics Statement

This work has been approved by the Research Ethics Committee of Golestan University of Medical Sciences (Gorgan, Iran) with the approval code of “IR.GOUMS.REC.1040.163.”

## Consent

Informed consent was written and signed by the patient.

## Conflicts of Interest

The author declares no conflicts of interest.

## Supporting information


**Data S1:** supporting Information.

**Data S2:** supporting Information.

**Data S3:** supporting Information.

## Data Availability

The data that support the findings of this study are available from the corresponding author upon reasonable request.
