# Supplementary figures and images for: Unilateral Gynecomastia and Primary Hypogonadism Following Brucellosis Infection: A Rare Case Report
Source: Clin Case Rep. 2025 Sep 8;13(9):e70880. doi: 10.1002/ccr3.70880 (PMC12417314; doi:10.1002/ccr3.70880)

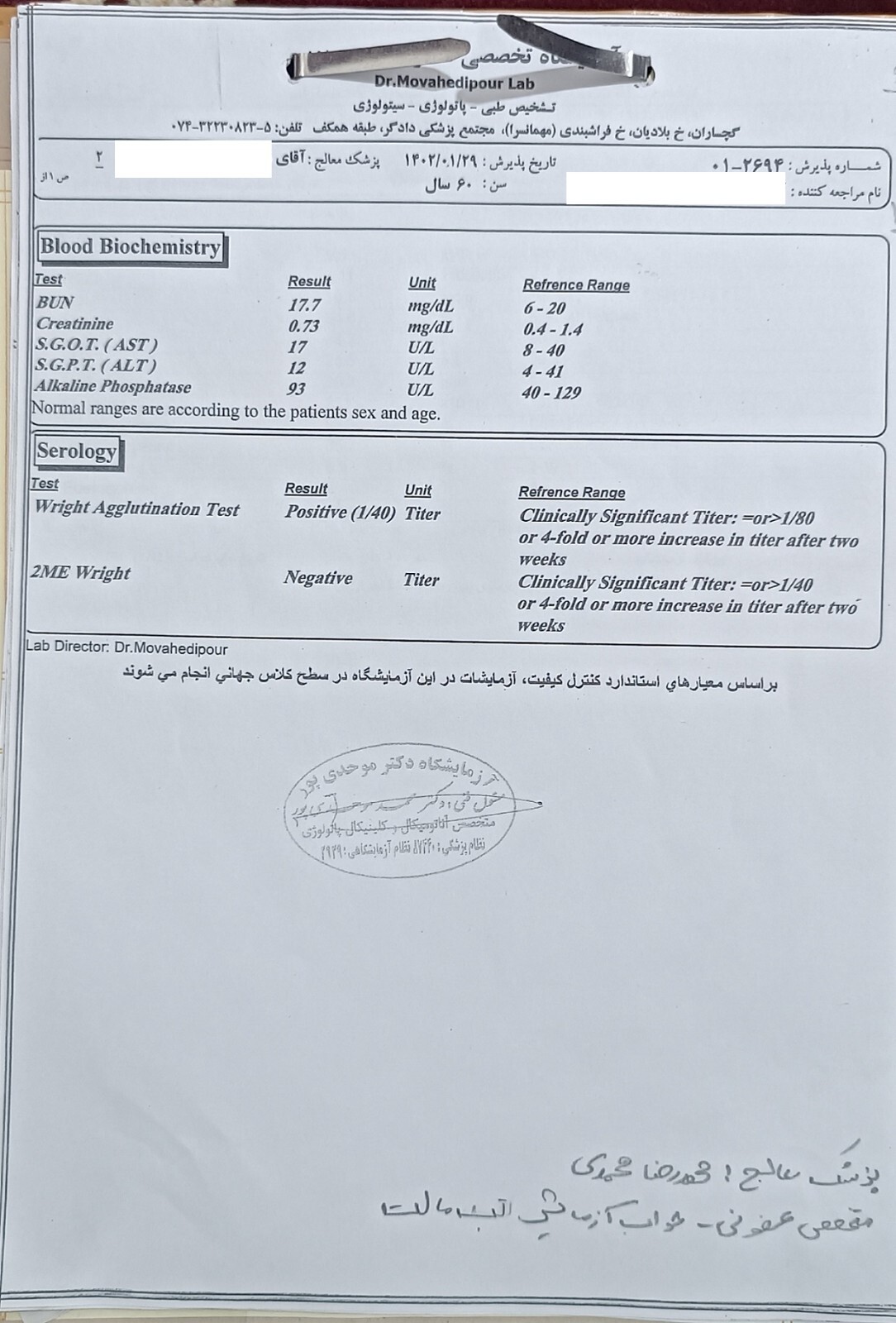

Supplement: Supplementary file 1 — Data S1: supporting Information. [file CCR3-13-e70880-s001.jpg]

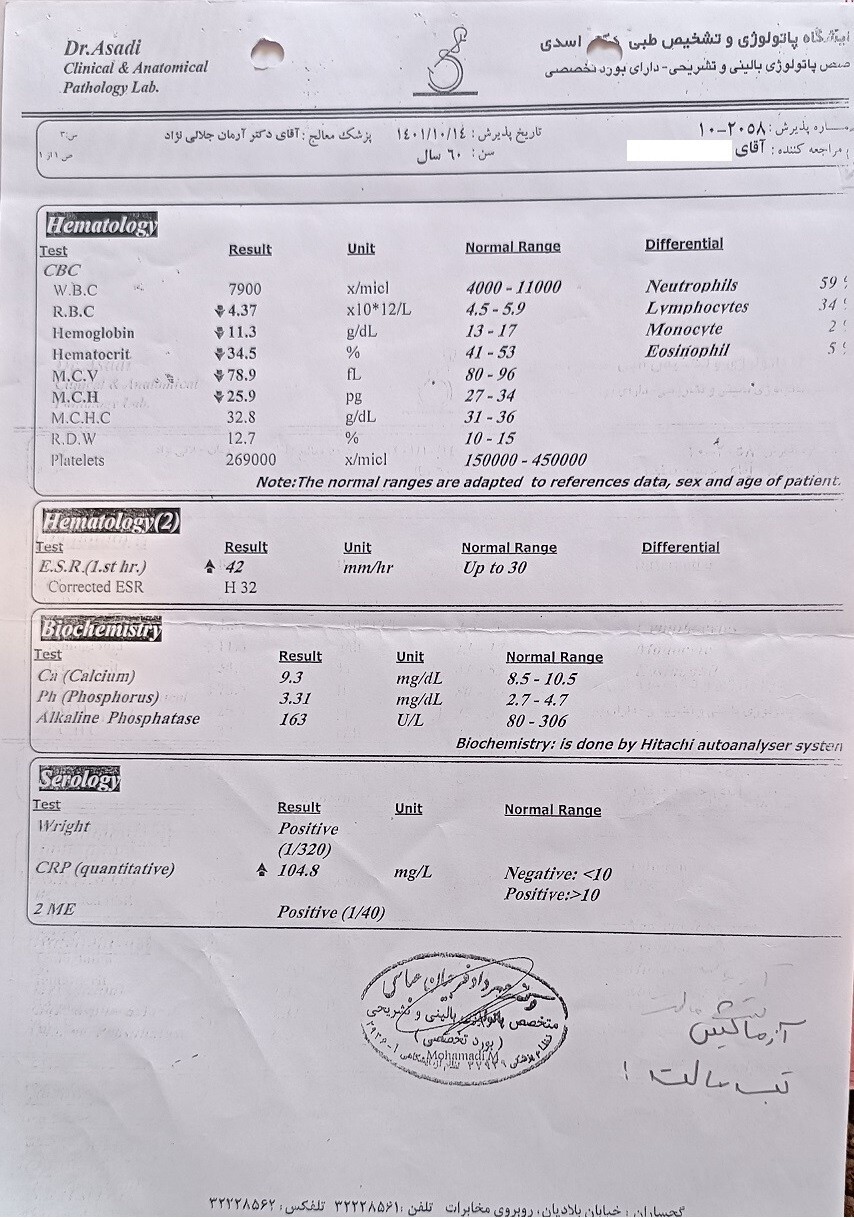

Supplement: Supplementary file 2 — Data S2: supporting Information. [file CCR3-13-e70880-s003.jpg]

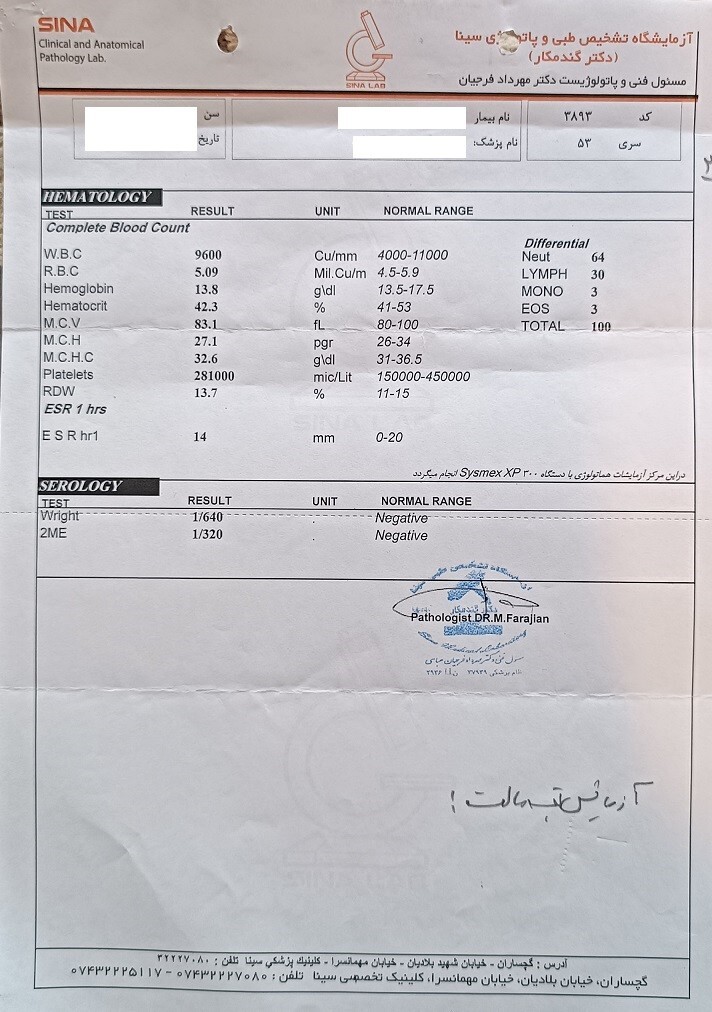

Supplement: Supplementary file 3 — Data S3: supporting Information. [file CCR3-13-e70880-s002.jpg]
